# Emergence of Coronavirus (COVID-19) Outbreak: Anthropological and Social Science Perspectives

**DOI:** 10.1017/dmp.2020.203

**Published:** 2020-06-24

**Authors:** Harshal B Sonekar, Manickam Ponnaiah

**Affiliations:** ICMR School of Public Health, ICMR-National Institute of Epidemiology, Chennai, Tamil Nadu, India

**Keywords:** emergency preparedness, public health, public health surveillance, public health practice, social networking

## Abstract

With the ongoing coronavirus (severe acute respiratory syndrome coronavirus-2, SARS-CoV-2), the entire community of health professionals is working to control disease and investing crores in vaccine development. The present discussion is to bring the focus on various social issues that emerge during outbreak and calls for equal attention as that of other health-care interventions. These issues are summarized in three categories: first, stigmatization due to lack of knowledge about the source of infection; second, speculations and their consequences around lack of knowledge about transmission; and finally, the concern regarding miscommunication during such a crisis. Most of these concerns emerge from press and social media coverage of the episode. The Ebola outbreak response is an example of how social scientists and anthropologists can work with other experts to solve questions of public health importance. Their approach toward the community with the objective to understand the sources, reasons, and circumstances of the infection will help to manage the current outbreak. In this context, we suggest collaboration of diverse scientific community to control and sensitize the people to tackle the misinformation in the affected and non-affected community during the outbreaks.

The latest outbreak of novel coronavirus infection (referred to as severe acute respiratory syndrome coronavirus-2 severe acute respiratory syndrome coronavirus-2, SARS-CoV-2) and the resulting disease as coronavirus disease 2019 (COVID-19)^1^ has been declared a public health emergency of international concern (PHEIC) by the World Health Organization.^2^ Around the world, 113,702 laboratory-confirmed COVID-19 cases (China = 80,924 cases) and 3140 deaths have been reported as of 10 March 2020.^3^ It has generated fear globally as it is tipped to be the next “pandemic”.^4^

Globally, in the recent past, several countries are witnessing outbreaks of emerging infectious diseases of zoonotic origin, Ebola, Nipah, and Zika to name a few. Countries have addressed these outbreaks systematically with a team of clinicians, health professionals, researchers, and policy-makers. In the context of COVID-19, we do not have sufficient knowledge about the spread of the infection in humans. While the scientific community is geared to examine the transmission from the basic science and epidemiological point of view, it requires involvement of other cross-cutting disciplines given the gamut of issues involved.

In this context, it may be useful to revisit some of the social consequences of the outbreak on the population. Rapid spread of this virus generated concerns among the public and health systems alike. Despite generation of sufficient knowledge about the actual spread of disease, there were issues with the assimilation of the same by different sections of the population. This could be due to the fact that the language used was predominantly technical in nature and, therefore, could not be understood by common people. Furthermore, there were no opportunities to seek and obtain clarification on the scientific terms. Therefore, it is necessary to highlight these issues that will merit systematic studies.

The first issue is that of reports of stigmatization due to lack of knowledge about source of SARS-CoV-2. Stigmatization is referred to as full denial of social acceptance.^5^ Several countries are reporting stigmatization, particularly toward the Asian population. Certain sections of the Asian population have been targeted based on their eating habits, which also includes consumption of wild animals. One has to look deeper to know the source of infection rather than just assuming the consumption of raw meat as the reason of contracting infection. For instance, we have been witnessing reports of “discrimination” in several public places toward Asian people across a few settings.^6^ This discrimination is ultimate result of lack of knowledge among people about the infection and its modes of transmission (person to person, animal to person, or through air). Such schema generate false images about people and formulates wrong perceptions which then are converted to xenophobia.^7–9^

Another issue that contributed to discrimination and stigmatization is in the context of migration. As a result of lockdown, migrants from poor communities did face discrimination due to their social status (for instance in the Indian context).^10^ Along with the lockdown, another terminology that added confusion was that of recommendation to maintain “social distancing.” In practice, it was meant to denote “physical distancing” to prevent person-to-person transmission through cough or secretions. However, it sounded as if people should stop communicating with each other.

Recently, Australia condemned the extent of xenophobia associated to COVID-19 and expressed support to people facing discrimination in several places.^11^ Hence, it is of serious concern and reiterates the importance of the need to dispel myths and concentrate on relying on available facts and generating new knowledge.

Second concern is regarding speculations and its consequences around lack of knowledge about transmission. At the early stage of the outbreak, and to some extent even now, the source of infection was attributed to the eating habits of “exotic” foods of animal origin. This knowledge is still inadequate and is based on clinical history by professionals and workers who are dealing with COVID-19 patients.^12^ In China though, the meat consumption practices historically were not prevalent but the insect eating practices goes back to 3000 years.^13^ According to Chinese custom, food is thought be the expression of “live fully” and is considered to be part of artistic culture and has sentimental connections to people. However, the same idea of food consumption in the Western part is more of scientific in nature.^13,14^ The lack of hygiene maintained in Wuhan market was depicted in such a way that it that could cast aspersions about people’s behavior without respecting their cultural heritage and practices. Food practices are indeed closely related to culture and are followed across several generations.

Finally, miscommunication is a concern during outbreak times, a little wrong communication can generate panic and fear among the people, and it can happen at any level. During such crises, the important faces, such as leaders at various levels, are looked upon for information. They need to have clarity about the disease transmission and correct prevention and control measures so that the messages conveyed to the population adequately.

Furthermore, communications within households and in the community are of importance. If not well-informed, then miscommunication among them can spread to a larger community. Therefore, all concerns related to infection and disease are to be disseminated and communicated well within the family members to overcome panic and anxiety. However, generating information around the source and emergence of the infection requires systematic in-depth investigation of family members, care givers, other social contacts, and stakeholders in the community, including observation of various settings.^15^ Such investigations require an empathetic approach and emotional involvement with the respondents by maintaining professional ethical etiquette.

If investigated, this may help in teasing out the right approach to communicate useful information toward controlling infection spread. Over-abundance of a mixed bag of information makes it difficult for the community to look for a credible information source and get reliable guidance whenever they need it.^16^ An “infodemic” is worse than an epidemic, because it causes fear and rumors to spread multiple times faster than the disease-causing agent.

To address these concerns, it may be useful to bring in researchers, who are experts in ethnographic studies (social scientists and medical anthropologists) and other social science methods. Taking a collective response from the affected people helps in building future strategies.^17^ This can be seen in previous studies on the Ebola virus. In 1997, Hewlett and Hewlett deployed the first anthropological interventions in Gabon, a place in central Africa where the Ebola outbreak occurred as an unknown entity. They provide the intense account of socio-cultural and political contexts of disease, which show the importance of anthropology as a tool of investigation in public health emergencies.^18^ In Ebola, even today, anthropological investigations help in identifying hitherto unknown modes of spread. For instance, nosocomial practices were documented to have led to reinfection in a hospital setting.^19^ In fact, organizations like “A group of Social Science and Humanitarian Action Platform” (combined group of health professionals including medical anthropologist) and “Ebola Response Anthropology Platform” has systematically addressed the issues of recent Ebola outbreak.^17^ We need more such collaborations, specifically in low and middle income countries (LMIC).

In addition, anthropologists are better interventionists in addressing issues around public health emergencies by becoming an integral part of the affected community. They can help build strategies by engaging with the community, religious leaders, influential members, decision-makers in the community, and other stakeholders to prevent miscommunication. It also helps by keen observation of the individual chain for transmission of diseases by deploying relevant methods and tools in contact tracing. Such interventions help in reducing risk of exposure and further health crises. People may feel confident to speak transparently of their issues and concerns and fears.

The above concerns are global and are not specific to any country. The sudden emergence of diseases and viruses can occur anywhere and anytime. Generating precise scientific knowledge in such situations from social science and medical anthropologists and health professionals is an essential need of health emergencies. Their approach toward the affected community/population in a humane manner with the objective to understand the ways and means of spread of infection and eventually to decipher the sources, reasons, and circumstances. This knowledge is essential to dispel misinformation during health crises. Hence, a first step in addressing the stigmatization is to generate appropriate knowledge about the COVID-19. This to be done on micro and macro level. To achieve this, collaboration among diverse scientific community is required to map out the preliminary strategies and to control ongoing outbreaks, and such preparation can help in a future crisis in such an outbreak. Second, there is a gap in which ideas about transmission is disseminated or assimilated from and among the public health experts to reach community as per their requirement. For example, in the United States, multiple media interviews by people suggest that their perception of coronavirus is just a flu. This will pose a challenge when people think their illness will not harm them as they relate it to the flu-like illness. Hence, it is important that the knowledge translation should be constructed in way that it will sensitize people and provide appropriate knowledge to tackle spread of rumors and misinformation in the affected and nonaffected communities. Finally, it is significant to address main-stream society and media regarding their responsibility during the public health emergency to spread core and authentic information in their neighborhood.
